# Physicochemical Equivalence and Quality Assessment of Various Brands of Gastro-Resistant Omeprazole Capsules in the Kumasi Metropolis

**DOI:** 10.1155/2022/7924600

**Published:** 2022-11-09

**Authors:** Yaa Asantewaa Osei, Elvis Oppong Boakye, Marcel T. Bayor, Frederick William Akuffo Owusu

**Affiliations:** Department of Pharmaceutics, Faculty of Pharmacy and Pharmaceutical Sciences, Kwame Nkrumah University of Science and Technology, Kumasi, Ghana

## Abstract

The proliferation of counterfeit and poor-quality drugs is a major public health problem, especially in developing countries such as Ghana where there are inadequate resources to effectively monitor their prevalence. Most of these drugs, which are counterfeited, are drugs, which are in high demand and will reap huge profits for the unscrupulous people who engage in such activities. The introduction of Omeprazole as one of the first-line therapies in the management of peptic and duodenal ulcers in the treatment guidelines of Ghana has resulted in many generics being introduced onto the market. The pharmaceutical quality of fifteen randomly sampled Omeprazole capsule brands in the Kumasi metropolis was assessed using the innovator brand as a comparator to confirm their suitability for patient use and to provide data for drug regulatory agencies in Ghana concerning poor quality omeprazole brands. All the sampled brands complied with the official specifications for identification with good primary and secondary packaging characteristics. Ninety-four (94%) of the sampled brands passed the uniformity of weight test. All the brands (*n* = 16) representing 100% passed the disintegration tests. Sixty percent (60%) of the sampled brands passed the drug content test. Ten brands (66.7%) met the specification for *in vitro* dissolution test. From *f*2 analysis, the dissolution profiles of only five brands (31%) were similar to that of the reference brand which indicated that they could be used interchangeably in clinical practice. Conclusively, ten out of the fifteen sampled brands were of good quality and only five could be used as a substitute for the innovator. Thus, regulatory agencies will need to strengthen their postmarket surveillance to ensure that generic brands of good quality are allowed onto the market.

## 1. Introduction

Good health is an integral part of achieving the World Health Organization's (WHO) sustainable development Goal 3 and thrives on complete health care. Complete health care can be achieved with good pharmaceutical care and quality medicines [1]. However, it is known that; substandard and counterfeit medicines have become a widespread problem in low and lower-middle-income countries [2–4]. It is estimated that 1 in 10 medical products in low- and middle-income countries is substandard or falsified. Substandard medicines are widespread and represent a threat to health [5].

Gastroduodenal conditions have emerged as one of the noncommunicable diseases (NCD) affecting adults in Ghana [6, 7]. In the United States, peptic ulcer disease affects approximately 4.6 million people annually, with an estimated 10% of the US population having evidence of a duodenal ulcer at some time. Peptic ulcer disease refers to painful sores in the lining of the stomach (gastric ulcer) or the first part of the small intestine (duodenal ulcer) [8]. The treatment of Peptic ulcer disease or gastroesophageal reflux disease is very effective with the combination of antibiotics, antiacids, and proton pump inhibitors [9].

Proton pump inhibitors are well known to be valuable in the treatment of gastric ulcers. Recent studies have shown that omeprazole is one of the most effective antisecretory drugs currently available. At 20–40 mg once daily, omeprazole provides cumulative healing rates of up to 100% after 4–8 weeks. In several comparative studies of omeprazole and ranitidine, similar healing rates in the acute treatment of gastric ulcers were seen though there was a tendency towards more rapid healing with omeprazole. A more recent multicenter study showed signs that more ulcers healed after 4 weeks with omeprazole, both 20 mg and 40 mg once daily than with ranitidine, 150 b.d. mg omeprazole was also superior to ranitidine concerning the relief of night-time pain and in gastric ulcer healing during concomitant therapy with nonsteroidal anti-inflammatory drugs. Omeprazole is therefore a valuable alternative in the modern treatment of gastric ulcers, being superior to histamine *H*2-receptor antagonist [10, 11].

One critical component of the health care system is generic drugs. They account for about 90% of dispensed prescriptions in the United States [5]. Generic drugs are known to contain equivalent amounts of the same active component(s) as their brand-name counterparts, but usually cost far less. Wide variations in prices of marketed omeprazole gastro-resistant capsules exist, particularly between generic products and proprietary ones (Innovator brand). Generic formulations of branded drugs offer therapeutic alternatives for physicians and the patients they treat [12, 13]. However, there is a likelihood of variations in the pharmaceutical quality of generic brands of omeprazole which can be attributed to several formulation factors. For example, differences in the quality of granule coating may be a source of variability in the in-vitro and in-vivo availability of omeprazole [12]. Packaging types have also been reported to be an additional factor significantly influencing formulation stability and performance. Wide variations in prices of marketed omeprazole capsules currently exist, particularly between generic products and proprietary ones (Innovator brand). [12, 14]. However, there are legitimate concerns about the efficacy of generic formulations and data show that cost savings to the patient or the healthcare system may not be as significant as expected. The Innovator brand of omeprazole is normally between 2–7 times high priced than the generic equivalents. Although generic medicines are more available than innovator brands in public and private health clinics, the innovator brands are usually purchased more than the generic brands [15]. The chemical structure and mechanism of action of both generic and innovator brands of omeprazole may be similar, but their effectiveness may be different due to the difference in quality and formulation characteristics. Omeprazole is the first-line antisecretory agent used in Ghana and over the years the proliferation of various brands in the Ghanaian market has been of concern. This, therefore, poses the need to continually assess the quality of these brands to protect health so that pharmaceutical companies do not take undue advantage of patients.

This study, therefore, seeks to assess the quality of various brands of omeprazole capsules with the innovator brand as a comparator.

## 2. Materials and Methods

Dipotassium hydrogen orthophosphate and potassium dihydrogen orthophosphate (UK Chemicals, Kumasi), Whatman filter papers (UK Chemicals, KNUST), Brands of Omeprazole capsules (Registered Pharmacies in Kumasi), and Pure omeprazole powder (Ernest Chemist Limited, Accra, Ghana). All reagents used were of analytical grade.

### 2.1. Methodology

#### 2.1.1. Sampling of Omeprazole Capsules from the Market

Sixteen (one innovator and fifteen generic) brands of Omeprazole capsules were purchased from registered retail Pharmacies within the Kumasi metropolis. Brands that were sampled had a shelf life of at least one (1) year remaining from the time they were purchased. The various brands were coded for research purposes and analysis was performed before product expiration dates. Capsules to be tested were selected randomly from their packages and tested according to methods outlined in the various Pharmacopeias.

#### 2.1.2. Packaging and Labelling Inspection

The primary and secondary packages, labels, unique identification marks, foils, expiry dates, and leaflet inserts were carefully examined for required information such as product name, manufacturer's address, manufacturing dates, batch numbers, and expiry date. The capsules to be analyzed were critically examined on both sides for unique markings and any alterations in physical appearance. The Food and Drugs Authority number was also checked on the product.

#### 2.1.3. Identification of Omeprazole Using Fourier Transmittance Infrared Spectroscopy (FTIR)

The test was run on each brand by placing a small quantity of crushed and powdered capsules of the specific brand on the FTIR spectrometer (PerkinElmer UATR two), disc and scanned within a wavelength of 4000–400 cm^−1^. Methanol was used to clean the FTIR disk after each brand was scanned to prevent contamination. The spectra obtained were then compared to that of the pure sample [16].

#### 2.1.4. Uniformity of Weight

Twenty (20) capsules from each brand were randomly selected for this test. The content of the capsule was removed without altering the integrity of the capsule shell, and the weight of the individual empty capsule shell was determined. The net weight of each capsule content was determined by subtracting the weight of each empty capsule shell from the initial capsule weight. This test was carried out for all the brands and the values obtained were recorded. The mean weight, standard deviation, and percentage standard deviation were subsequently determined (British Pharmacopoeia 2014, 2014) [17, 18].

*(1) Disintegration Test*. The disintegration time for all the brands was determined according to the USP method [12] using a disintegration apparatus (Ewerka, Type ZT 3/1, GmbH Heusenstamm, Germany Nr 68318). The experiment was conducted at 37°C using 0.1 M HCl for two hours followed by phosphate buffer pH 6.8 as the immersion fluid. Six Capsules were randomly chosen from a brand and placed in each of the six cylinder-shaped tubes of the basket rack of the disintegration apparatus individually. The base of the basket rack was placed such that it was not less than 15 mm below the surface of the disintegration medium. Three determinations were conducted and the mean disintegration time was established for each brand [12, 17, 18].

#### 2.1.5. Calibration Curve

A stock solution of Omeprazole with a concentration of 0.1% w/v was prepared by dissolving 0.1 g of pure omeprazole powder in a small volume of phosphate buffer of pH 6.8 and made up to 100 ml with the phosphate buffer. The following concentrations: 0.006, 0.009, 0.012, 0.015, 0.018, 0.025, and 0.028% w/v were then prepared from the stock solution. The absorbance of these solutions was then determined by ultraviolet spectrophotometry using a UV spectrophotometer (2440 Double beam SHIMADZU Corporation, JAPAN) at a wavelength of 303 nm. A calibration curve showing the relationship between concentration and absorbance was plotted and the equation and correlation values of the curve were obtained from the scatter plot.

#### 2.1.6. Determination of Drug Content (Assay)

One randomly selected capsule from each brand was crushed and dissolved in 100 ml of phosphate buffer 6.8. The resulting mixture was filtered using a Whatman number 1 filter and made up to 100 ml using a phosphate buffer of pH 6.8 in a volumetric flask. The final solution was diluted suitably, and the absorbance was measured at 303 using phosphate buffer as blank on a UV spectrophotometer (2440 Double beam SHIMADZU Corporation, JAPAN). This procedure was repeated ten times for each batch and the average value was used in subsequent calculations [16, 19, 20].

#### 2.1.7. In Vitro Drug Release (Dissolution Test)

The dissolution studies on the various brands of omeprazole were conducted using the USP dissolution apparatus I (basket apparatus), (Ewerka, Type DT6, GmbH Heusenstamm, Germany Nr 68045). 900 ml of 0.1M HCL was used as the dissolution medium for the first hour followed by phosphate buffer pH 6.8 maintained at 37°C ± 2°C with an agitation speed of 100 rpm. 10 ml of samples in a dissolution medium were withdrawn and filtered at different time intervals of 10, 20, 30, 40, 50, and 60 minutes, respectively. An equal volume of a fresh medium having the same temperature was replaced at each time to maintain the sink condition in the vessel. The withdrawn samples were filtered with Whatman filter paper (No. 1) and suitably diluted. The diluted filtrates were analysed on a UV spectrophotometer (2440 Double beam SHIMADZU Corporation, JAPAN) at a wavelength of 303 nm. Using the equation obtained from the calibration curve, the concentration of omeprazole in samples was calculated and the percentage release values were computed. A plot of the mean cumulative percentage of drug release against time was established [12, 16, 19].

#### 2.1.8. Statistical Analysis

Graph Pad Prism (version 5) was used to analyze the brands statistically using a *T*-test. The research was conducted using OM 16 as the standard. All the other brands were subsequently compared to the standard to obtain the *p* values. The *p* value helps to give a clue as to whether the means of the paired samples are significantly different.

## 3. Results and Discussion

### 3.1. Product Information Details

Information obtained from the primary and secondary packaging as well as visual inspection of capsules from the various brands has been summarized in Table 1. 95% of the sampled brands were imported products from other countries with only one being manufactured locally. This suggests the high prevalence of foreign brands of omeprazole capsules in the country. 33.3% of the brands were not registered with the FDA at the time of the study (Table 2). All the brands had information leaflets inserted with adequate data, 60% of the brands were formulated as enteric-coated/normal-release capsules and 40% were delayed-release formulations as indicated by the manufacturers. All the brands were aluminium foil blister-packed except OM4 which was plastic blister-packed. On inspection of the packs and labels on the primary and secondary containers of the different brands, they all met the British Pharmacopeia specifications for packaging and labelling of omeprazole capsules [17].

### 3.2. Identification and Authentication of Active Ingredient (Omeprazole) in the Sampled Brands

The identification test is one of the crucial initial tests that need to be performed whenever there is a basis for it. The identity of the active ingredient in the sampled brands and the reference sample (pure sample) needs to be ascertained before further experimental work could be done.

**Figure 1:**
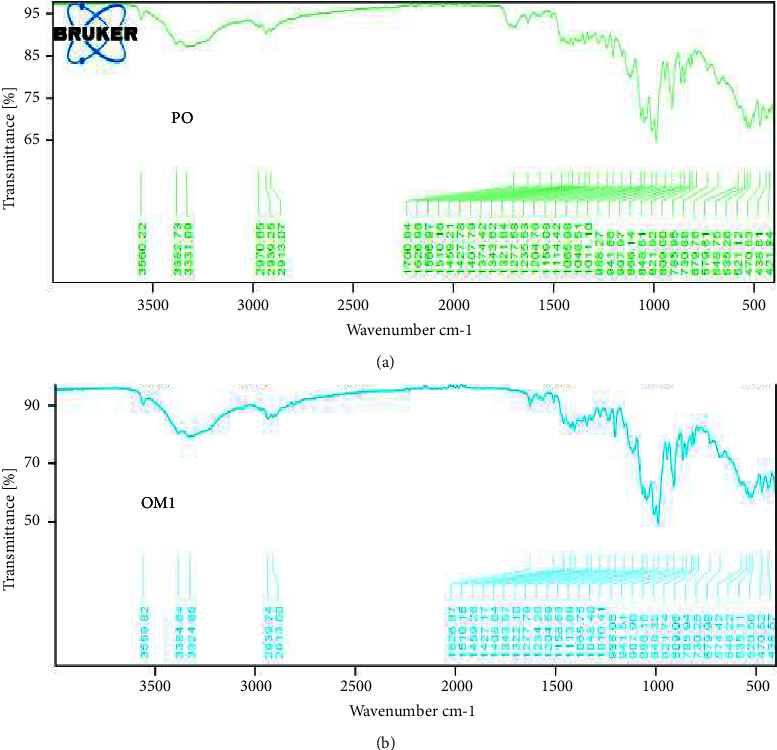
FTIR spectra of (a) pure omeprazole (PO) and (b) OM1.

Chemically, Omeprazole as a molecule is divided into four fragments. The first fragment is a substituted pyridine ring, the second is a benzimidazole and the third is a C-SO-C bridge. The fourth fragment consists of various methoxy groups. NH stretching in the infrared spectrum is usually visible as a medium-intensity band in the range between 3500 and 3200 cm^−1^. However, the NH group from the benzimidazole fragment of the molecule is a potent proton donor, which aids in hydrogen bonds with several types of proton acceptors inferring that NH stretching frequency would undergo redshift for the strength of the established hydrogen bond. Besides, the formation of hydrogen bonds invariably broadens the NH vibrational band. Therefore, the NH stretching vibration can be found in the infrared spectrum of Omeprazole as broadband near 2930 cm^−1^. This was observed in all the sampled brands as well as the reference pure sample (Figure 1). Again, the Reference IR spectrum of pure omeprazole powder is characterized by features such as characteristic absorption bands C-H stretching at 3057 cm^−1^ and the absorption bands at 2984 cm^−1^ and 2972 cm^−1^ assigned to the stretching vibrations of aromatic CH_3_ due to the methoxy group. There is also a characteristic band stretching vibration at 2854 cm^−1^ specific to the symmetric C-H_3_ methoxy group (-CH3). At 1629 cm^−1^ there appears a characteristic band indicative of C-C stretching and CH deformation of pyridine and benzimidazole groups. Absorption bands at 1423 cm^−1^, 1181 cm^−1^, and 1013 cm^−1^ are attributable to the pyridine group. High-intensity bands characteristic of S=O stretching vibrations occur at 978–381 cm^−1^ [21, 22]. The IR spectrum obtained for the pure omeprazole powder and the various brands exhibited absorption bands around the aforementioned wavenumbers (Figure 1). Thus, the identity of the pure omeprazole powder and the sampled brands was authenticated.

### 3.3. Uniformity of Weight

An indispensable imputes for the capsule is the requirement for a constant dose of drug among individual capsules within a batch. Minute variations amongst individual capsules are acceptable and the maximum variations are well enshrined as standards in the various pharmacopoeias. The uniformity of weight test is one of the tests which are performed to ensure (to some extent) constant dosing among capsules to mitigate the commonness of overdosing or underdosing. There appears to be a direct correlation between the variation in the weight of individual capsules and the corresponding variation in the drug content [16, 23]. The amount of granules placed in the body of a capsule will ultimately determine the weight of the encapsulated product [24]. Sampled brands of omeprazole capsules had weights of less than 300 mg. Therefore, for a batch of such capsules to pass the weight uniformity test, not more than two of the individual weight of the capsules should deviate from the average weight by more than the percentage deviation of ±10%. Moreover, none of the tablets is to deviate by more than twice (±20%) the permissible percentage deviation [17]. From the results obtained in Table 3, fifteen (15) of the sampled brands of omeprazole capsules passed the uniformity of weight test with one (1) brand failing the test, three capsules from the batch deviated from the mean weight by more than 10%.

### 3.4. Disintegration Test

An essential component in the release of the active pharmaceutical ingredient (omeprazole) from the capsule is the disintegration time. Disintegration precedes dissolution and therefore gives an idea (to a lesser extent) of how fast the drug is being released into the immersion medium and made available for dissolution. According to the British Pharmacopoeia [17]; the disintegration of gastro-resistant capsules occurs when there is no palpable firm core of the test sample after it is operated in a buffer of pH 6.8 for a maximum duration of sixty (60) minutes after the start of the disintegration experiment. The ability of a gastro-resistant capsule to pass the disintegration test primarily depends on the nature and integrity of the polymer used in designing the coating for the capsule pellets. Other factors such as the particle size of pellets, and the temperature of the fluid in the disintegration apparatus can contribute significantly to the omeprazole capsules passing or failing the test [12]. All the brands sampled passed the disintegration test (Table 3), indicating that the polymer and the method used in designing and manufacturing the gastro-resistant pellets were good and will release their contents within the stipulated time.

### 3.5. Drug Content

As stated by Okorie et al. [12]; out of the eleven brands of omeprazole capsules, which were sampled and tested in Nigeria, two showed an unacceptable quantity of active contents against the label claims. Based on this research finding and combined with the fact that an estimated 30% of the medicinal products on sale for consumption in many countries in Africa are counterfeit and substandard [15], it gives rise to concern about the quality of omeprazole capsules in Ghana, because Ghana and Nigeria share our drug market due to the Economic Community of West African States (ECOWAS) trading systems.

According to the standards of the USP, upon assay of omeprazole in the capsules, the active content should lie between 90%–110% of the label claim. Considering the results obtained (Table 3), nine brands had active content that conformed to the USP standards. Six (6) brands (OM2, OM4, OM5, OM9, OM10, and OM15) had values that fell outside the acceptable range. The brands OM2 and OM4 had a percentage content of active ingredients below the lower limit of 90%. These brands may produce a subtherapeutic effect when used by patients. Possible reasons why brands OM2 and OM4 failed the tests could include inaccuracy in weighing the active ingredient, lack of effective mixing during the granulation, and incorporation of less amount of the active ingredient during the formulation. On the other hand, brands OM5, OM9, OM10, and OM12 had a percentage content more than the upper limit of 110%. This will result in these brands producing an overdose of omeprazole in the patient coupled with increased incidences of adverse effects. These brands having higher percentage drug content could be due to; inaccuracy in weighing the active ingredient, lack of effective mixing during the granulation, and incorporation of excess amount of the active ingredient during the formulation. The high drug content may imply noncompliance to current Good Manufacturing Practices and possible formulation errors [12]. Any drug formulation that contains an overdose or lower dose can still be considered substandard.

### 3.6. Dissolution Test

#### 3.6.1. Percentage Drug Release

The oral route continues to offer the major means of drug administration in the treatment of diseases. The efficacy of the oral dosage form depends on the disintegration and dissolution of the dosage unit in the desired fluids and the subsequent absorption into the systemic circulation. The rate at which this occurs is therefore very crucial and worth studying [20, 25]. Dissolution testing is utilized in differentiating the influence of manufacturing variables such as mixing effect, granulation procedure, and excipients type and can subsequently be used to predict product behaviour *in vivo* and in some cases to determine bioequivalence and assure interchangeability [26].

**Figure 2:**
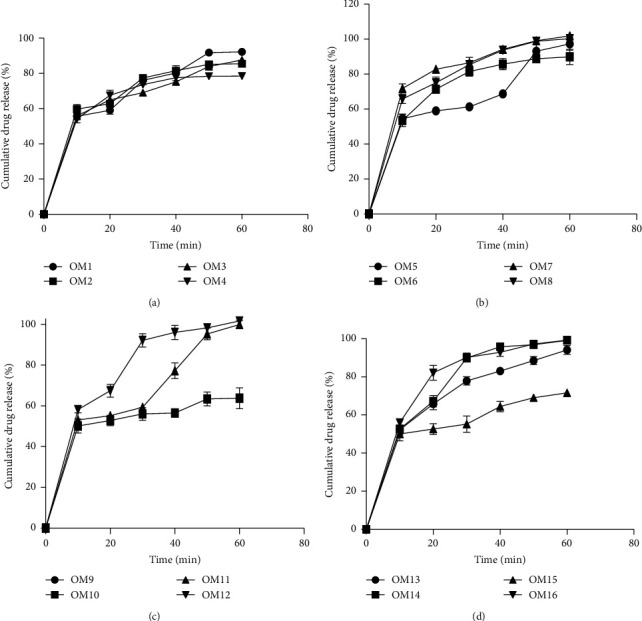
(a) Dissolution profiles of OM1, OM2, OM3, and OM4, (b) dissolution profiles of OM5, OM6, OM7, and OM, (c) dissolution profiles of OM9, OM10, OM11, and OM, and (d) dissolution profiles of OM13, OM14, OM15, and OM1.

According to the monographs in the United States Pharmacopoeia, each of the brands tested for dissolution must have not less than 75% of the prescribed or stated amount of the active content in the solution within 30 minutes in a phosphate buffer of pH 6.8. From the results all the brands except OM3, OM4, OM5, OM11, and OM15 (Figures 2(a)–2(d)) passed the USP general specification standard for the dissolution rate test. Variations in the manufacturing processes account for the major reason why these brands failed the dissolution test. The dissolution test gives the closest In vivo-In vitro correlation thus failure of this test connotes that the brand may not elicit the desired effect when taken by patients.

#### 3.6.2. Similarity Factor (Model-Independent Method)

In comparisons of dissolution profiles, in particular, to ensure resemblance in product results, the statutory interest is to know how comparable the two dissolution curves are. If the two profiles are identical, *f*2 = 100. The FDA has established a government *f*2 standard of 50–100 to show the resemblance between two dissolution profiles.

In the dissolution profile comparisons, using the *f*2 values calculated only five brands OM7, OM8, OM9, OM12, and OM14 had values above 50% with OM8 recording 96.57% (Table 4), which was closest to 100. FDA has set a public standard of *f*2 value of 50–100 to indicate similarity between two dissolution profiles. This implies that only five brands of the sampled omeprazole products were similar to the innovator brand.

For product substitution, only five brands OM7, OM8, OM9, OM12, and OM14 can be considered because they have biopharmaceutical equivalence.

## 4. Conclusion

This study has revealed that most of the brands of Omeprazole capsules marketed in Ghana are foreign brands. OM16 which was used as the standard passed all the pharmacopoeia and nonpharmacopeial tests. All the sampled brands contained omeprazole but 38% did not contain the specified amount. Only five brands OM7 OM8, OM9 OM12, and OM14 can be used as a substitute for the standard (OM16).

## Figures and Tables

**Table 1:** Product information details.

Code	Batch number	Manufacturing date	Expiry date	Registration status	Registration number
OM1	270127	11/17	10/20	Not registered	Nil
OM2	7K18	11/17	10/20	Registered	FDA/SD. 183–2121
OM3	M7125	11/17	10/20	Registered	FDA/SD. 173–10801
OM4	172025	Nil	07/19	Not registered	Nil
OM5	OP6001	01/18	12/20	Registered	Nil
OM6	H330	05/17	11/19	Registered	Nil
OM7	H-04004	09/16	08/19	Registered	FDA/SD. 183–7456
OM8	OP7019	07/17	06/20	Not registered	Nil
OM9	C-010006	11/17	10/20	Not registered	Nil
OM10	06709	Nil	07/19	Not registered	Nil
OM11	OP8007	02/18	01/21	Registered	Nil
OM12	CC125	03/18	02/21	Registered	FDA/SD. 193–7581
OM13	HN4547	Nil	09/19	Not registered	Nil
OM14	UNC008	Nil	09/20	Registered	FDA/SD 183–9587
OM15	18008	Nil	01/20	Registered	Nil
OM16	YCFW/0010	Nil	04/20	Registered	Nil

**Table 2:** Physical assessment of sampled brands.

Brands	Presence of leaflet	Nature of capsule	Nature of packaging
OM1	Leaflet present	Enteric-coated/normal release	Aluminium foil blister
OM2	Leaflet present	Enteric-coated/normal release	Aluminium foil
OM3	Leaflet absent	Delayed-release	Aluminium foil
OM4	Leaflet present	Enteric-coated/normal release	Blister packaging
OM5	Leaflet present	Delayed-release	Aluminium foil
OM 6	Leaflet present	Enteric-coated/normal release	Aluminium foil
OM 7	Leaflet present	Enteric-coated/normal release	Aluminium foil
OM 8	Leaflet absent	Delayed-release	Aluminium foil
OM 9	Leaflet present	Enteric-coated/normal release	Aluminium foil
OM 10	Leaflet present	Enteric-coated/normal release	Aluminium foil
OM 11	Leaflet present	Delayed-release	Aluminium foil
OM 12	Leaflet present	Delayed-release	Aluminium foil
OM 13	Leaflet present	Enteric-coated/normal release	Aluminium foil
OM 14	Leaflet present	Enteric-coated/normal release	Aluminium foil
OM 15	Leaflet present	Enteric-coated/normal release	Aluminium foil
OM 16	Leaflet present	Enteric-coated/normal release	Aluminium foil

**Table 3:** Disintegration time and drug content of sampled brands.

Brands	Average disintegration time (minutes) (*n* = 6)	Average % drug content (*n* = 10)
OM 1	02 : 46 ± 0.043	97.09 ± 0.05^*∗*^
OM 2	02 : 52 ± 0.063	86.25 ± 0.13^^^
OM 3	02 : 58 ± 0.013	108.06 ± 0.25^*∗*^
OM 4	02 : 57 ± 0.013	79.18 ± 0.11^^^
OM 5	02 : 06 ± 0.009	128.12 ± 0.21^^^
OM 6	03 : 30 ± 0.006	91.99 ± 0.06^*∗*^
OM 7	02 : 58 ± 0.008	100.66 ± 0.19^*∗*^
OM 8	02 : 45 ± 0.004	107.06 ± 0.22^*∗*^
OM 9	02 : 40 ± 0.067	118.20 ± 0.08^*∗*^
OM 10	02 : 21 ± 0.088	112.93 ± 0.03^^^
OM 11	03.51 ± 0.009	97.09 ± 0.42^*∗*^
OM 12	03 : 37 ± 0.024	102.39 ± 0.30^*∗*^
OM 13	03 : 09 ± 0.033	101.72 ± 0.15^*∗*^
OM 14	02 : 50 ± 0.066	95.46 ± 0.43^*∗*^
OM 15	03 : 27 ± 0.089	123.27 ± 0.45^^^
OM 16	01 : 37 ± 0.089	103.96 ± 0.05^*∗*^

^*∗*^Sample passed the content test; ^^^sample failed the content test.

**Table 4:** Similarity factor for sampled brands.

Brand	Similarity factor (*f*2)
OM 1	27.93
OM 2	27.11
OM 3	21.63
OM 4	20.25
OM 5	22.17
OM 6	29.22
OM 7	73.64+^*∗*^
OM 8	98.06+^*∗*^
OM 9	51.36+^*∗*^
OM 10	6.61
OM 11	23.75
OM 12	67.03+^*∗*^
OM 13	27.44
OM 14	52.54+^*∗*^
OM 15	10.17

+^*∗*^Samples with high *F*2.

## Data Availability

The data used to support the findings of this study are included in the article and also available from the corresponding author upon request.
